# Electrocoagulation treatment of high saline oily wastewater: evaluation and optimization

**DOI:** 10.1016/j.heliyon.2020.e03988

**Published:** 2020-06-05

**Authors:** Forat Yasir AlJaberi, Shaymaa A. Ahmed, Hasan F. Makki

**Affiliations:** a Chemical Engineering Department, College of Engineering, Al-Muthanna University, Al-Muthanna, Iraq; b Chemical Engineering Department, College of Engineering, University of Baghdad, Baghdad, Iraq

**Keywords:** Chemical engineering, Environmental chemical engineering, Chemical reaction engineering, Electrochemical engineering, Adsorption, Water treatment, Real oily saline wastewater, TDS, TSS, HCO3, CL, Ca, Electrocoagulation reactor, Optimization

## Abstract

The present work provides to treat real oily saline wastewater released from drilling oil sites by the use of electrocoagulation technique. Aluminum tubes were utilized as electrodes in a concentric manner to minimize the concentrations of 113400 mg TDS/L, 65623 mg TSS/L, and the ions of 477 mg HCO_3_/L, 102000 mg Cl/L and 5600 mg Ca/L presented in real oily wastewater under the effect of the operational parameters (the applied current and reaction time) by making use of the central composite rotatable design. The final concentrations of TDS, TSS, HCO_3_, Cl, and Ca that obtained were 93555 ppm (17.50%), 11011 ppm (83.22%), 189ppm (60.38%), 80000ppm (22%), and 4200 ppm (25%), respectively, under the optimum values of the operational parameters (1.625 Amps and 40 min). In spite of the low removal percentages of some pollutants, the present study proved the ability of this novel designed reactor for treating high saline real oily wastewater in accordance with the operational parameters. This prove the capability of the use of it as a pre-treatment of other conventional methods.

## 1 Introduction

Ecological pollution caused by oily wastewater produced from several industrial activities, such as the drilling oil sites, petroleum refineries, and petrochemical industries constitutes, is a serious threat to the environment [1]. The drilling oil sites contain numerous types of pollutants that may be categorized into organic (petroleum hydrocarbons) and inorganic compounds such as oil content, COD, BOD, TOC, turbidity, TDS, TSS, cyanide, ammonia and heavy metals [2]. They differ in their concentration according to the locations of the crude oil wells and their depth (1–40000 ppm) [3, 4]. The percentage of oily wastewater amounts presented in produced crude oil ranging from 0.4 to 0.6 [5].

High saline oily wastewater that containing high amounts of total dissolved solids (TDS) and total suspended solids (TSS), is discharged from several industrial activities into the water resources and soils without using any pre-treatment which causes severe environmental calamities [6, 7].

In order to overcome this kind of pollution, several techniques had been employed for treating saline oily wastewater [2, 8, 9, 10] such as the conventional technologies like gravity sedimentation and dewatering, but the biological treatment methods are limited in application because the high salinity wastewater (>1% salt) which leads to loss of cell activity [6]. The performance of any treatment method is characterized by the removal efficiency, the time required to accomplish the treatment, secondary contamination produced, and the cost of construction, operating and maintenance [3], but they possess some notable drawbacks that minimize their efficiencies and applications [2, 11]. Therefore, an effective treatment method should be employed to overcome the disadvantages of using conventional treatment technologies and recovery of water under contingent limits [11].

Among conventional treatment techniques, the electrochemical technique had achieved an essential gain in oily wastewater treatment due to its unique advantages such as simple design and operation, cost-effective, and high efficiency [8, 12]. This technique contains several categories such as electro-oxidation, electro-Fenton and electrocoagulation [13].

Electrocoagulation is a type of electrochemical processes which considered as an effective method for the treatment of oily wastewater because it possesses many merits such as simple apparatus, less reaction time, no chemicals added and low consumption of energy and electrodes [2, 4, 14] beside that, its ability to be employed as a pre-treatment method or in hybrid systems with other treatment methods such as adsorption and ionic exchange processes [1, 5]. In contrast to other methods of wastewater treatment, the electrocoagulation technique requires only simple equipment and less time of treatment, but the mechanism of oil removal from oily wastewater is complicated owing to their physicochemical parameters [3].

**Figure 1:**
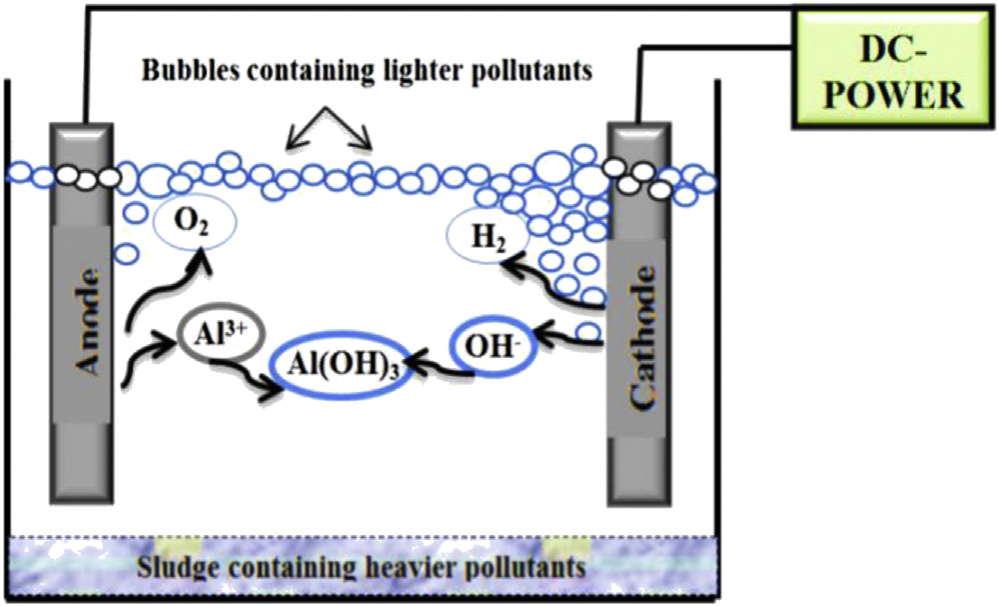
Schematic representation of the electrocoagulation process.

The Electrocoagulation mechanism depends on the redox reactions and the deposition process taking place in the reactor as a result of the passing of electric current through the electrodes that are usually made of aluminum and/or iron (Figure 1).

Metal cations, such as Al^3+^ or Fe^2+^ are released due to the dissolution of the anode, while the hydrogen gas and hydroxyl ions (OH^−^) are generated at the cathode [3, 8]. Electro-coagulants, such as Al(OH)_3_, are formed due to the chemical reactions that occur between different ions (Eqs. (1), (2), (3), (4), and (5)) [8].

(1) Al_(S)_ ⇒ Al^3+^_(aq)_ + 3e^−^

(2) 2H_2_O ⇒ O_2_ + 4H^+^ + 4e^−^

(3) 2H_2_O + 2e^−^ ⇒ H_2(g)_ + 2OH^−^_(aq)_

(4) Al^3+^ +3OH^−^ ⇔ Al(OH)_3_

(5) n Al(OH)_3_ → Al_n_(OH)_3n_

The electro-coagulants formed during the electrocoagulation process, i.e. Al(OH)_3_, possess a larger capability than the chemical coagulants for eliminating contaminants from different kinds of wastewaters. Furthermore, they have a larger size than that the size of the chemical coagulants which cause a minimum solubility of products within a specific range of pH, and as a consequence, this will lead to an easy separation [3].

There are numerous designs of electrocoagulation reactors depending on their electrodes metal, shape, and configuration, the mode of operating (batch and continuous), and AC/DC current supplied [14].

The design of the electrocoagulation reactor utilized in this study was invented by the author (Forat Yasir AlJaberi) and described in details in another study [14]. In brief, the reactor involves three aluminum tubes arranged in a monopolar-parallel-concentric mode where the inner and the outer tubes had designed as the anode while the middle one was selected as the cathode.

Based on the literature review, there are several studies concerning the treatment of oily wastewater using electrocoagulation technique [1, 5], but there is no previous one study utilized the present design of the electrocoagulation reactor for the treatment of real saline oily wastewater. According to literature review, several parameters had been investigated by other studies such as pH, conductivity, the distance between electrodes, electrolytes, etc. The present study aims to treat real oily wastewater, therefore pH, conductivity and electrolyte concentration were not taken into consideration but their values had measured as natural. This study intends to employ this novel reactor for minimizing the concentrations of 113400 mg TDS/L, 65623 mg TSS/L, and the ions of 477 mg HCO_3_/L, 102000 mg Cl/L and 5600 mg Ca/L presented in real high saline oily wastewater discharged from crude oil drilling site located in Basra-Iraq under the influence of the most effective operational variables (the applied current and reaction time) by applying the central composite rotatable design.

## 2 Materials and experimental work

### 2.1 Chemicals

Real saline oily wastewater was collected from a drilling site located in West Qurna 1/Basra-Iraq. Its characterization are shown in Table 1 where the collected samples had been preserved using polypropylene containers at 4 °C to be treated then by a batch electrocoagulation reactor.

**Table 1:** Characterization of real saline oily wastewater.

Parameters	Value
TDS (mg/L)	113400
TSS (mg/L)	65623
HCO_3_ (mg/L)	477
Cl (mg/L)	102000
Ca (mg/L)	5600
Oil content (mg/L)	523
TOC (mg/L)	34023
pH	6.5
Conductivity (ms/cm)	126

At the end of each run, the analytical determination of TDS, TSS, HCO_3_, Cl and Ca ions in the treated samples was carried out according to the standard methods for the examination of water and wastewater [15].

### 2.2 Apparatus

The present study utilized an electrocoagulation reactor containing three aluminum tubes arranged in a concentric manner. The outer and the inner tubes had been designed to work as the anode electrode while the tube placed in between them had been used as the cathode electrode. The physical composition of these tubes (Table 2) was analyzed in previous study by the authors [16] using EDS test (Oxford instrument-X-act) which shows that these tubes contain different compounds that may affect the behavior of the treatment process as will showing later.

**Table 2:** Characterization of aluminum tubes.

Compounds	Al	Mg	Fe	Si	C
Weight %	85.88	0.97	0.06	0.76	12.33

**Figure 2:**
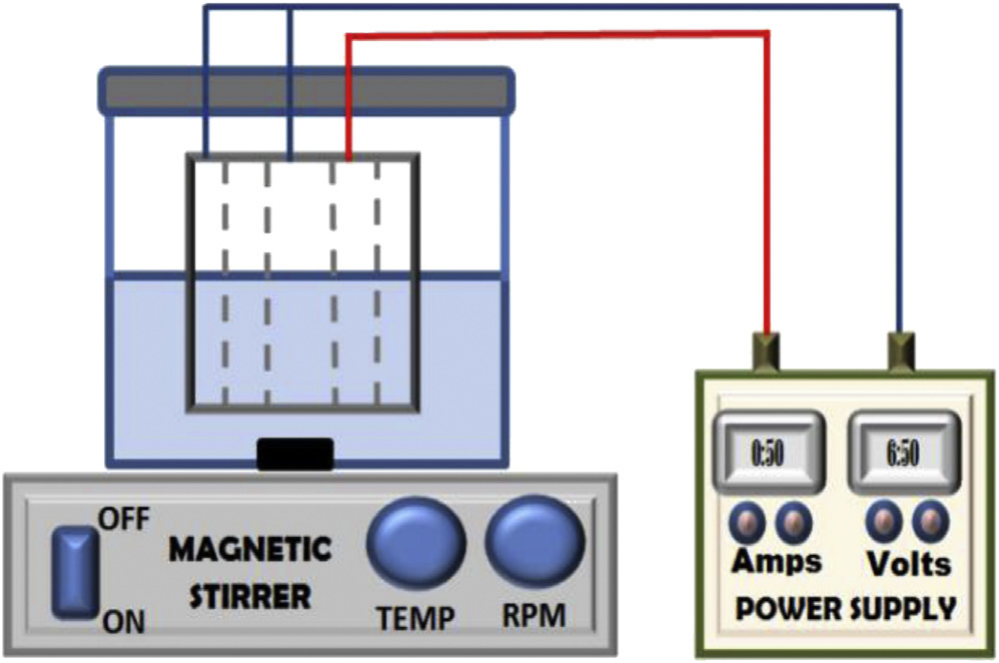
Schematic of the electrocoagulation reactor.

The designed experiments had been carried out in the batch electrocoagulation reactor shown in Figure 2. The monopolar-parallel concentric electrodes have a total effective area equaling 285cm^2^ where the immersed height of these electrodes was 4cm. These electrodes had connected to a regulated digital DC power supply (0–30 volt and 0–5 Amps) manufactured by SYADGONG company (model 305D-China), where the voltage was maintained constant while the applied current was adjusted to the desired value and the duration of treatment was conducted as designed. The polluted sample was completely agitated using an electromagnetic stirrer (ALFA company: HS-860; 0–1000 rpm) during the treatment process at a constant agitation speed of 200 rpm.

### 2.3 Experimental design

A statistical technique method of surface response methodology, central composite design (CCD), and a statistical program (Minitab-17) were performed to design the experiments and to predict the impact of the operational variables on the studied responses, the interaction between variables and the nonlinear relationships with responses.

The mathematical correlation between responses and the operational variables is estimated by fitting them into second-order polynomial equation (Eq. (6)) where the indicator of the quality of the obtained model is the higher value of the regression coefficient (R^2^) obtained as well as the results of the analysis of variance (ANOVA) [16, 17]:

(6) $$\text{Y}={\text{B}}_{\text{0}}+\sum_{\text{i}=1}^{\text{q}}{\text{B}}_{\text{i}}{\text{X}}_{\text{i}}+\sum_{\text{i}=1}^{\text{q}}{\text{B}}_{\text{ii}}{\text{X}}_{\text{i}}^{2}+\sum_{\text{i}}\sum_{\text{j}}{\text{B}}_{\text{ij}}{\text{X}}_{\text{i}}{\text{X}}_{\text{j}}+\text{ε}$$

Where Y represents the studied responses; X_1_, X_2_, to X_q_ are the operational variables; q is the total number of the operational variables; B_o_ is an intercept regression constant, B_i_ is the linear regression effect, B_ii_ is the quadratic regression effect and B_ij_ is the interaction regression effect; **ε** is a random error.

For electrodes, the distance among them is constant due to the novel design of using triple concentric tubes. Thereby, the use of this invented reactor tends the author to choose the most important parameters that extremely impact the treatment process in such type of electrochemical technique. Depending on previous studies, the ranges of the operational parameters (applied current and reaction time) were taken to study all the variations of the behavior of the studied responses as possible [3, 8].

In this study, the operational variables; applied electric current (X_1_) and reaction time (X_2_); had been studied according to the ranges of (0.5–2.0 Amps) and (10–40 min) respectively. Where the mixing speed was fixed at 200 rpm for all experiments.

The total number of experiments (N) designed according to CCD was obtained using the following equation (Eq. (7)):

(7) N = q^2^ + 2q + n

Total 11 runs with 4 factorials, 4 axial and 3 center points (n: number of replicates) were suggested by design experts to optimize the studied responses, that is, TDS and TSS removal. The experimental ranges and levels of the operational variables are listed in Table 3 while the design of experiments according to RSM using CCD with the aid of Minitab program is shown in Table 4.

(8) $${\text{X}}_{\text{coded}}=\frac{\frac{{\text{X}}_{\text{Real}}-{\text{X}}_{\text{Center}}}{{\text{X}}_{\text{Center}}-{\text{X}}_{\text{min}}}}{\sqrt{\text{q}}}$$

and the rotatability [α=(2^q^)^0.25^] equals ±1.414.

**Table 3:** Experimental ranges and levels of the operational variables.

Real Variables (Xi)	Coded Variables				
	Axial point (-α)	Lower level	Centre point	Upper level	Axial point (α)
	-1.414	-1	0	+1	1.414
X1: Applied current (Amps.)	0.5	0.72	1.25	1.78	2.0
X_2_: Reaction time (min.)	10	14	25	36	40

Where the real values of the operational variables (X_Real_) were estimated from the following equation (Eq. (8)).

**Table 4:** Design of experiments.

Run	X_1_: Coded value	X_2_: Coded value	X_1_: Applied Current (Amps.)	X_2_: Reaction time (min)
1	-1	-1	0.72	14
2	-1	1	0.72	36
3	1	-1	1.78	14
4	1	1	1.78	36
5	0	-1.414	1.25	10
6	0	1.414	1.25	40
7	-1.414	0	0.50	25
8	1.414	0	2.00	25
9	0	0	1.25	25
10	0	0	1.25	25
11	0	0	1.25	25

The responses of TDS and TSS removal efficiencies (Y) were obtained using Eq. (9) as follows:

(9) RE% = [(C_i_-C)/C_i_] × 100

Where C_i_ and C are the initial and final values of pollutants (mg/l). Each experiment had been repeated with three replications to ensure the accuracy and the average mean values were reported.

The adsorption process occurring through the electrocoagulation treatment technology is depending on the formation of the electro-coagulants as a result of redox reactions which is enormously monitored by the reaction time and the applied current according to Faraday's law shown in Eq. (10) which determines the theoretical amount of metal ions released from the electrodes [3].

(10) m_theo_ (g) = I. t. M / Z. F

Where I is the applied current in (Amps.), t is the reaction time in (second), M is the molecular weight of electrodes metal in (g/mol.), Z is the number of electrons presented in the reaction (for Al is 3), and F is Faraday's constant (96485.34 Columb/mol.).

## 3 Results and discussion

**Figure 3:**
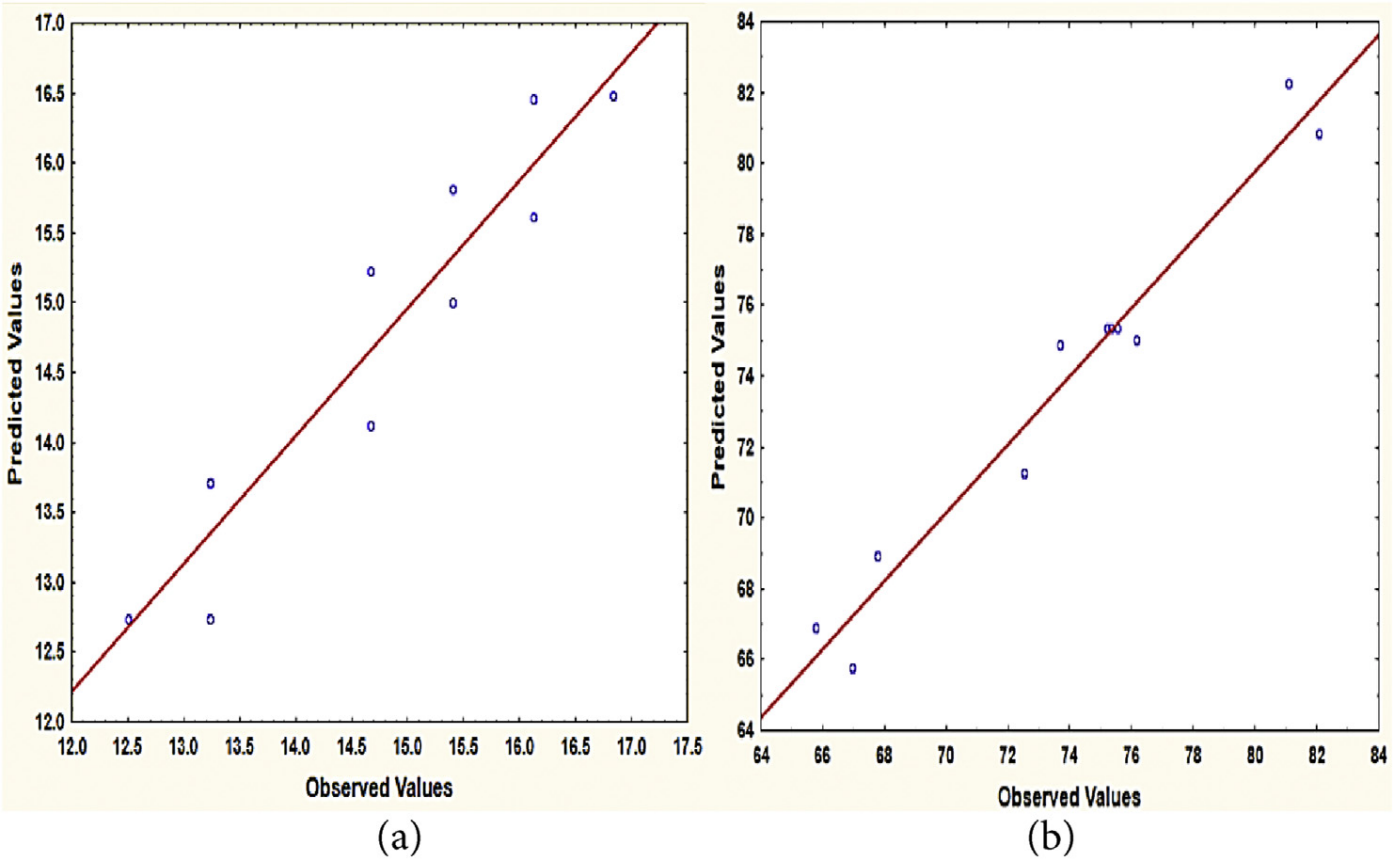
Predicted and actual value for (a) TDS removal % and (b) TSS removal %.

Table 5 provides the obtained results of the experimental and predicted values of TDS and TSS removal efficiencies, and the theoretical consumption of the tubular electrodes. The observed TDS and TSS removal values vary between 12.50-16.84% and 65.77–82.08% which are in good agreement with their predicted values as revealed in Figure 3.

**Table 5:** Results of the studied variables.

Operational variables			m_theo_ (g)	Actual values		Predicted Values	
Run	X_1_: Applied Current (Amps.)	X_2_: Reaction time (min)		TDS removal (%)	TSS removal (%)	TDS removal (%)	TSS removal (%)
1	0.72	14	0.058	14.67	66.94	14.12	65.80
2	0.72	36	0.143	15.40	76.15	15.01	75.02
3	1.78	14	0.143	16.12	72.51	15.62	71.29
4	1.78	36	0.355	16.84	82.08	16.49	80.88
5	1.25	10	0.070	14.67	67.76	15.23	68.94
6	1.25	40	0.280	16.12	81.08	16.46	82.24
7	0.50	25	0.070	13.23	65.77	13.71	66.89
8	2.00	25	0.280	15.40	73.69	15.82	74.92
9	1.25	25	0.175	12.50	75.56	12.74	75.37
10	1.25	25	0.175	12.50	75.33	12.74	75.37
11	1.25	25	0.175	13.23	75.21	12.74	75.37

### 3.1 Mathematical correlation of the studied responses

Based on experimental results, the mathematical correlations (Eqs (11) to (14)) shown in Table 6 were developed in terms of coded and real factors revealing the interactions between the operational variables to obtain TDS and TSS removal efficiencies:

**Table 6:** Coded and real mathematical correlations for the studied responses and their regression coefficients.

Responses	terms	Mathematical correlations	R^2^ (Adjusted)	R^2^
TDS Removal: Y_TDSR_	Coded	${{\text{Y}}_{\text{TDSR}}}^{\text{coded}}\text{\%}=12.743+1.489\;{\text{X}}_{1}+0.875\;{\text{X}}_{2}+2.023\;{{\text{X}}_{1}}^{2}+3.104\;{{\text{X}}_{2}}^{2}-0.005\;{\text{X}}_{1}{\text{X}}_{2}$ (11)	0.8272	0.9136
	Real	${{\text{Y}}_{\text{TDSR}}}^{\text{exp}}\text{\%}=24.180-7.574\;{\text{X}}_{1}+0.648\;{\text{X}}_{2}+3.596\;{{\text{X}}_{1}}^{2}+0.0138\;{{\text{X}}_{2}}^{2}-0.0004\;{\text{X}}_{1}{\text{X}}_{2}$ (12)		
TSS Removal: Y_TSSR_	Coded	${{\text{Y}}_{\text{TSSR}}}^{\text{coded}}\text{\%}=75.366+5.676\;{\text{X}}_{1}+9.405\;{\text{X}}_{2}-4.465\;{{\text{X}}_{1}}^{2}+0.227\;{{\text{X}}_{2}}^{2}+0.18\;{\text{X}}_{1}{\text{X}}_{2}$ (13)	0.9220	0.9610
	Real	${{\text{Y}}_{\text{TSSR}}}^{\text{exp}}\text{\%}=46.33+24.79\;{\text{X}}_{1}+0.373\;{\text{X}}_{2}-7.93\;{{\text{X}}_{1}}^{2}+0.001\;{{\text{X}}_{2}}^{2}+0.016\;{\text{X}}_{1}{\text{X}}_{2}$ (14)		

### 3.2 Analysis of variance (ANOVA)

The analysis of variance (ANOVA) is essential for estimating the quality of the model fitted; therefore, it was performed as a tool of analysis for fitting the function to the data that may conduct misleading results then the required models cannot be described adequately.

Table 7 provides the analysis of variance for TDS and TSS removal efficiencies where the values of Prob (P) less than 0.050 mean that the model terms are significant, but the regression model is classified as insignificant when their values are larger than 0.100. In the present study, The values of F are 10.56 and 24.65 for TDS and TSS, respectively, which indicate that the estimated models are significant. Moreover, the high values of the regression coefficients for both responses implies that these models are significant and are in reasonable agreement with the adjusted R^2^ values. Thereby, the correlations of TDS and TSS removal efficiencies will be as follows (Eqs. (15) and (18)) after omitting effects that having (P-Value) larger than 0.05 (**Bolded** values in Table 7):

(15) Y_TDSR_^coded^ % = 12.73 + 0.980 X_1_ + 1.981 X_1_^2^ + 3.103 X_2_^2.^

(16) Y_TDSR_^exp.^ % = 24.19–7.57 X_1_ + 3.596 X_1_^2^ + 0.0138 X_2_^2^

(17) Y_TSSR_^Coded^ % = 75.366 + 5.676 X_1_ + 9.405 X_2_ - 4.465 X_1_^2^

(18) Y_TSSR_^exp.^ % = 46.330 + 24.790 X_1_ + 0.373 X_2_ - 7.930 X_1_^2^

**Table 7:** ANOVA results for TDS and TSS removal efficiencies (Bolded numbers mean insignificant effect).

Source	Degree of Freedom	Sum of squares	Mean square	F-Value	P-Value
**TDS removal**					
**Model**	10	23.5.014	0.4061	10.57	0.011 (significant)
**X**_**1**_	1	4.4385	4.4385	10.93	0.021
**X**_**2**_	1	1.5317	1.5317	3.77	**0.109**
**X**_**1**_^**2**^	1	5.7776	5.7776	14.23	0.013
**X**_**2**_^**2**^	1	13.5941	13.5941	33.47	0.002
**X**_**1**_**∗X**_**2**_	1	0.0000	0.0000	0.00	**0.994**
**TSS removal**					
**Model**	10	284.197	2.2158	24.65	0.002 (significant)
**X**_**1**_	1	64.4146	64.4146	29.07	0.003
**X**_**2**_	1	176.8828	176.8828	79.83	0.0003
**X**_**1**_^**2**^	1	28.128	28.128	12.69	0.016
**X**_**2**_^**2**^	1	0.0726	0.0726	0.033	**0.863**
**X**_**1**_**∗X**_**2**_	1	0.0324	0.0324	0.015	**0.908**

Considering the obtained results of ANOVA analysis, it can be concluded that these models revealed the effective status of the electrocoagulation process and it can be performed to remove pollutants from oily wastewater.

### 3.3 Effect of the applied current

**Figure 4:**
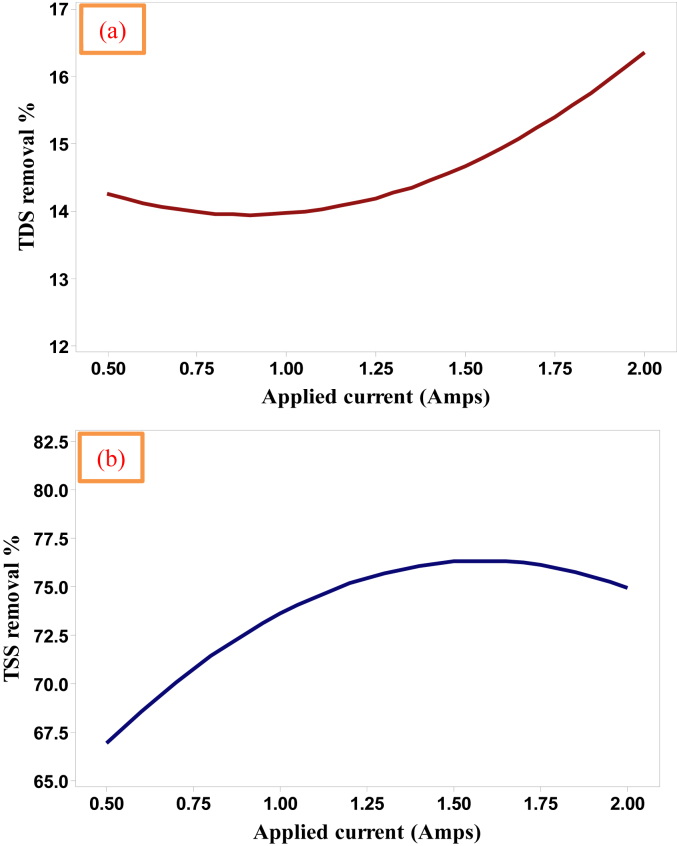
The effect of the applied current on the removal efficiencies of (a) TDS and (b) TSS of 113400 mg TDS/L, 65623 mg TSS/L, and the ions of 477 mg HCO3/L, 102000 mg Cl/L and 5600 mg Ca/L real saline oily wastewater (reaction time = 25 min).

**Figure 5:**
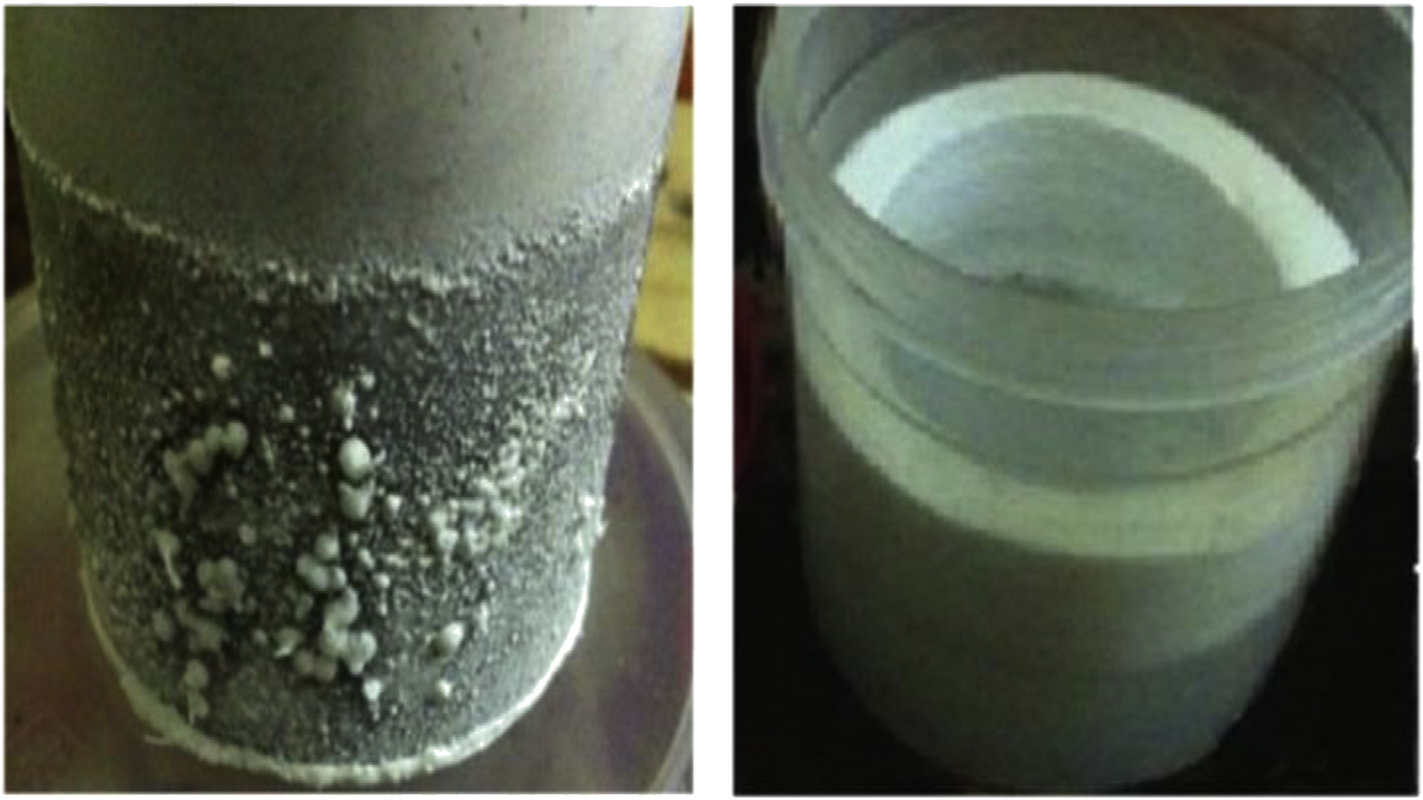
The aerated foams formed and salts transportation into the cathode surfaces during the treatment of 113400 mg TDS/L, 65623 mg TSS/L, and the ions of 477 mg HCO3/L, 102000 mg Cl/L and 5600 mg Ca/L real saline oily wastewater (reaction time = 25 min).

The impact of applied current on the studied responses is particularly vital because the electro-coagulants generation and gas bubbles releasing rates will extremely affect the rate of flocs formation and this is depending on the applied current; therefore, it should be considered in each process of electrocoagulation treatment because it control the amount of aluminum ions discharged from electrodes and the release of gases bubbles and as a consequence the formation of flocs [18]. According to the obtained results provided in Table 5, Figure 4 explains the behavior of TDS and TSS removal efficiencies with the applied current along the period of the mean range of the reaction time. The results shown in Table 5 revealed that the removal efficiencies of TDS and TSS raised from 13.23% and 65.77% at 0.5 Amps to about 15.40% and 73.69% at 2.0 Amps, respectively [8], which means that the final TDS and TSS concentrations in the treated water had declined from 113400 and 65623 mg/L to 95940 and 17267 mg/L, respectively. The interpretation of this behavior can be referred to the fact, that is, an increase of the applied current will reduce more of TDS due to the transportation process of the dissolved salts into the cathode surfaces (Figure 5) [6]. Moreover, TSS pollutant reduction occurred due to the influence of the adsorption process and the floatation of lightweight pollutants towards the surface of the solution as aerated foams (Figure 5) as a result of the effect of fine bubbles released at both electrodes [1]. As observed, an increase of the concentration of TDS and TSS was occurred due to the uncontrolled releasing of different ions from the electrodes that composite of different compounds (Table 1) as a result of redox reactions occurring through the electrocoagulation treatment [8] where these salts stilling in a suspended status neither adsorbed nor floated [3]. The behavior of these responses shown in Figure 5 are similar to that states by AlJaberi for the removal of oil content and turbidity [8].

### 3.4 Effect of reaction time

**Figure 6:**
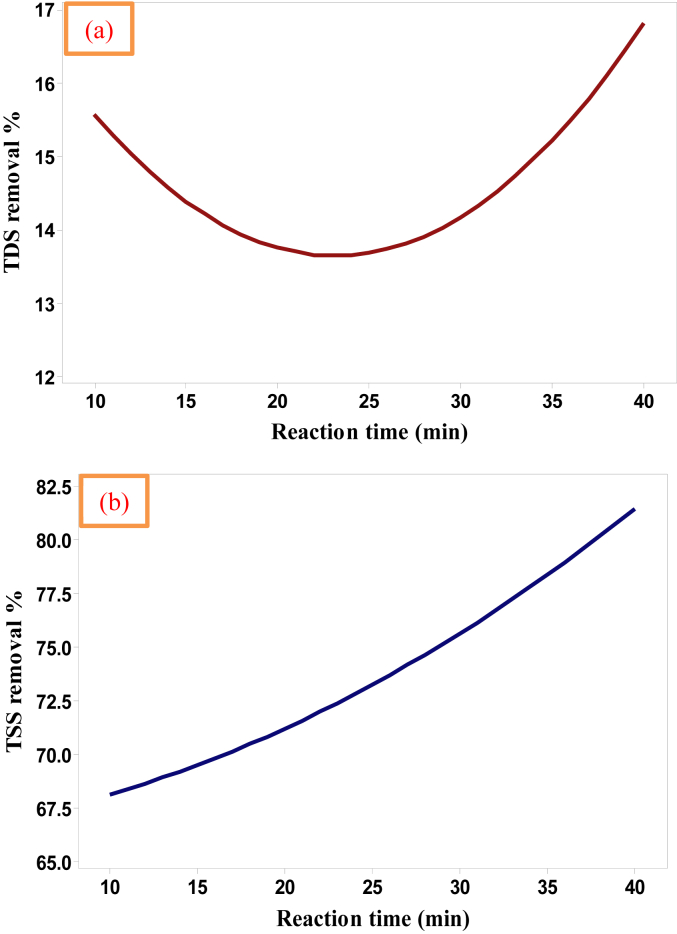
The effect of reaction time on the removal efficiencies of (a) TDS and (b) TSS of 113400 mg TDS/L, 65623 mg TSS/L, and the ions of 477 mg HCO3/L, 102000 mg Cl/L and 5600 mg Ca/L real saline oily wastewater (the applied current = 1.25 Amps.).

Electrolysis time is an important parameter for the formation of adequate quantity of various ions from electrodes which are required for the generation of adsorbents, such as Al(OH)_3_ in case of aluminum electrodes, as well as the discharging of gases bubbles from both electrodes which are essentially provided more assistance to carry the destabilized pollutants toward the surface of the solution by floatation which aid the removal of them. For TDS removal, it decreased along the period of the experiment until it reaches the mean range of the reaction time due to the huge amount of gas bubbles released in the first period of the experiment which impacts the electrodeposition process of the dissolved salts on the surface of the cathode then TDS removal tends to raise as the operation time increases. Meanwhile, the concentration of TSS was sharply minimized as the operation time increases in the case of the mean range of the applied current [6] due to the positive effect of the flotation process of floating suspended solids TSS because of their lightweight in comparison to the dissolved solid TDS. The impact of this variable (10–40 min) on the electrocoagulation removal of TDS and TSS was inspected and it is explained in Figure 6 according to the obtained results in Table 5.

As noted in Figure 6, when the reaction time increased from 10 min to 40 min, the removal efficiencies of TDS and TSS increased. Table 5 explained that TDS and TSS removal increased from 14.67% and 67.76% at 10 min to about 16.12% and 81.08% at 40 min, respectively by employing the present novel electrocoagulation reactor. Therefore, the higher the reaction time the higher removal efficiencies of these pollutants from the contaminated solution due to the significant actions of adsorption and desorption processes occurred throughout the electrocoagulation cell as the electrolysis time extended [8]. In contrast with the previous parameter, the removal of TDS had minimized at the beginning due to the uncontrolled releasing of different ions and gases from the electrodes as a result of redox reactions which impact the electrodeposition of the dissolved salts, while TSS removal was not altered in its behavior along the period of the experiment which means that the applied current could be optimized less than the ultimate range in order to obtain higher removal efficiencies of the studied responses as will explained in the next item.

### 3.5 Optimization of operating parameters

**Figure 7:**
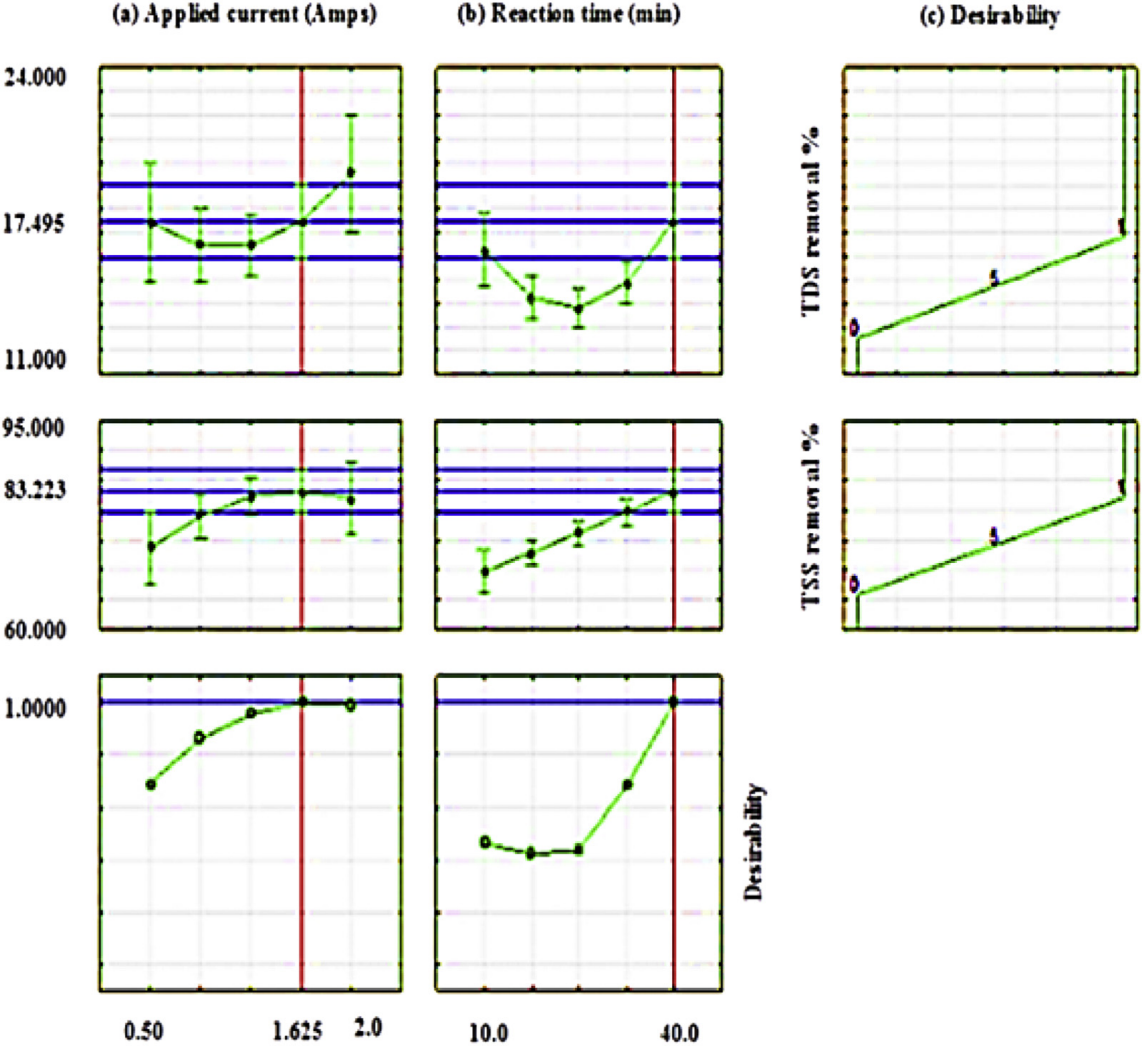
The optimum values of the operational variables (a) Applied current; (b) Reaction time and (c) the validated values of the studied responses for the treatment of 113400 mg TDS/L, 65623 mg TSS/L, and the ions of 477 mg HCO3/L, 102000 mg Cl/L and 5600 mg Ca/L real saline oily wastewater.

The optimum values of the operational variables were obtained by using a statistical software program (Minitab-17). Figure 7 explains the results of the D-optimization measurement where the composite desirability (D) equals 1. The optimum values of the applied current and the reaction time had been obtained as 1.625 Amps and 40 min, respectively, which attained 17.50% and 83.22% of TDS and TSS removal efficiencies, respectively. Where the obtained results of optimization are similar to that stated by the authors in a previous study for the removal of oil content and turbidity from oily wastewater [8]. This means that the present design of the electrocoagulation reactor could be used to remove different pollutants from oily wastewater under the optimum values of the operational variables designed. Under the optimum conditions of the operational variables, oil content and ions of HCO_3_, Cl and Ca had been declined from 523, 477, 102000 and 5600 mg/L to 82.460, 189, 80000 and 4200 mg/L, i.e. 60.38%, 22% and 25% respectively.

As observed in Figure 7, the increase in the applied current will give a slight increase in the TDS removal but TSS removal will be tended to minimize; therefore, the optimum values of the operational variables should be taken into consideration to attain the highest removal efficiencies and cost-effective process.

**Figure 8:**
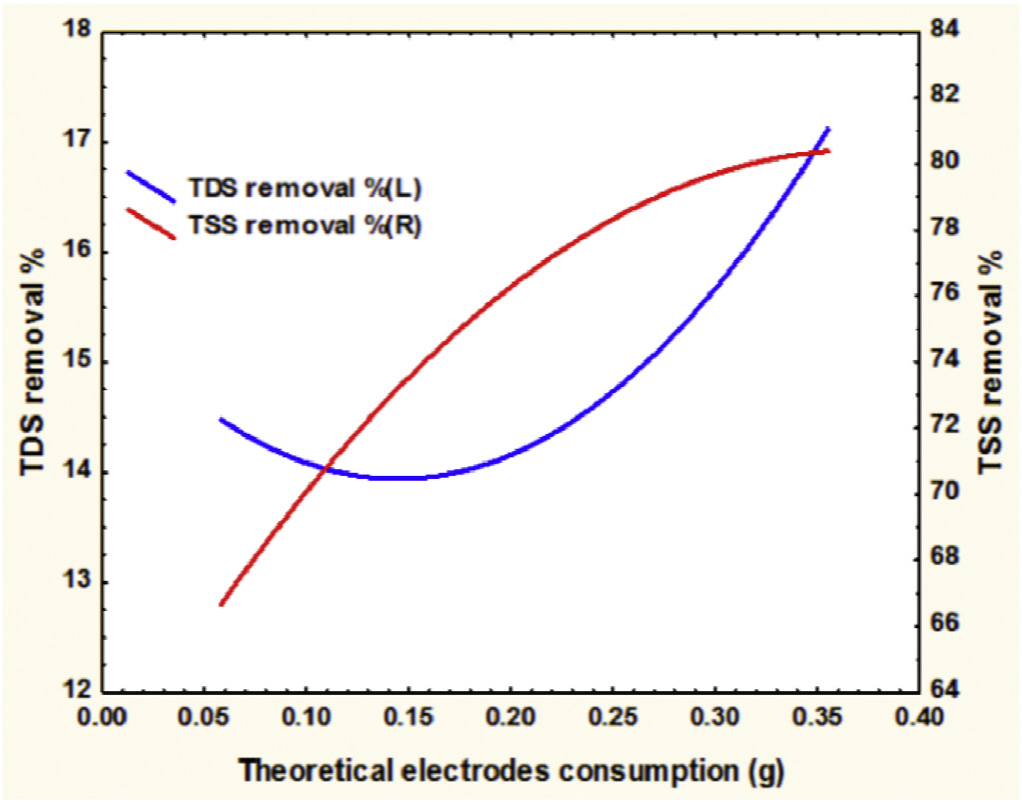
TDS and TSS removal efficiency vs. the theoretical consumption of electrodes for the treatment of 113400 mg TDS/L, 65623 mg TSS/L, and the ions of 477 mg HCO3/L, 102000 mg Cl/L and 5600 mg Ca/L real saline oily wastewater.

The behavior of TDS and TSS removal efficiencies with the theoretical consumption of electrodes is clearly explained in Figure 8 which is similar to the behavior of these responses with the studied variables because they are directly proportional with the theoretical consumption of the tubular electrodes according to Eq. (10).

As noticed in Figure 8, an increase of electrodes consumption will provide more of different ions as the applied current is passing through the electrodes along the periods of the experiments. At a low value of the consumption of the electrodes, TDS removal was minimized because gas bubbles had minimized or prevented the formation of the electro-coagulants but that will be more effective in floating of the suspended solids and maximizing TSS removal in a consequence. Therefore, the continuous increment of electrodes consumption will assist the removing of pollutants until it reaches the optimum values of the operating parameters.

## 4 Conclusion

The present study investigates the treatability of an invented electrocoagulation reactor for the treatment of 113400 mg TDS/L, 65623 mg TSS/L, and the ions of 477 mg HCO_3_/L, 102000 mg Cl/L and 5600 mg Ca/L real saline oily wastewater discharged from drilling oil sites (West Qurna 1/Basra-Iraq). The novel reactor attained good removal efficiencies of these pollutants by decreasing their initial concentrations to 93555 ppm (17.50%), 11011 ppm (83.22%), 189 ppm (60.38%), 80000 ppm (22%), and 4200 ppm (25%), respectively under the optimum values of the operational variables (1.625 Amps and 40 min). The mathematical correlations for the studied responses were estimated with a reasonable agreement. The analysis of variance ANOVA and the higher regression coefficients proved the satisfactory adjustment of the second-order polynomial model.

## Declarations

### Authors contribution statement

F.Y. AlJaberi: Conceived and designed the experiments; Performed the experiments; Analyzed and interpreted the data.

S.A. Ahmed: Analyzed and interpreted the data; Wrote the paper.

H.F. Makki: Conceived and designed the experiments; Contributed reagents, materials, analysis tools or data.

### Funding statement

This research did not receive any specific grant from funding agencies in the public, commercial, or not-for-profit sectors.

### Competing interest statement

The authors declare no conflict of interest.

### Additional information

No additional information is available for this paper.
